# Primary omental torsion (POT): a review of literature and case report

**DOI:** 10.1186/1749-7922-6-6

**Published:** 2011-01-26

**Authors:** Jacopo Andreuccetti, Cecilia Ceribelli, Ottavia Manto, Massimo Chiaretti, Paolo Negro, Domenico Tuscano

**Affiliations:** 1 Department of General Surgery and Organ Transplantation, "Sapienza" Università di Roma, Umberto I Policlinico di Roma, Rome, Italy

## Abstract

Eitel first described omental torsion in 1899, since then, fewer than 250 cases have been reported. Although omental torsion is rarely diagnosed preoperatively, knowledge of this pathology is important to the surgeon because it mimics the common causes of acute surgical abdomen. For this reason, in the absence of diagnosed preexisting abdominal pathology, including cysts, tumors, foci of intra-abdominal inflammation, postsurgical wounds or scarring, and hernial sacs, omental torsion still can represent a surprise. Explorative laparotomy represents the diagnostic and definitive therapeutic procedure. Presently laparoscopy is the first choice procedure.

## Introduction

Omental Torsion (OT) is a condition in which a pedicle of the omental apron twists on its longer axis to such an extent that its vascularity is compromised. Eitel [1] in 1899 first reported a case of omental torsion unassociated with a hernia. Since that time many reports have appeared in the literature, notably that by Morris [2] in which 164 authentic cases of torsion of the omentum were gathered from 1905 to 1930. OT may be Primary Omental Torsion (POT) because a mobile, thicken segment of omentum rotates around a proximal fixed point in the absence of any associated or secondary intra-abdominal pathology. Morris [3] reported that, while POT may occur at any age, it most frequently occurs between 30 and 50 years (83 cases, 52.5%), the males are more commonly affected than females (ratio of 2:1), and that the Secondary Omental Torsion (SOT) is mostly associated with predisposing pathology (50.3%). Morris [2], Adam [3] and Barcia & Nelson [4] emphasized the fact that the hernias were of the right inguinal variety, were scrotal type, of long duration, easily reducible, and that they almost invariably contained omentum. In this condition, patients suffering from recurring abdominal pain may have temporary twists of the omentum. The "omental ball" and the omental fibrotic thickenings occasionally found, result from these recurring attacks of incomplete OT. The chronic changes occasionally found in the omentum in acute (complete) OT [5], seem to substantiate the occurrence of the recurring type of OT. A certain number of OT are caused by inflammatory foci within the abdominal cavity, which produce an inflammation by contiguity in the neighbouring omentum. This may be true in cases of mild or subsiding appendicitis or cholecystitis in which the original focus subsides, but the changes induced in the omentum persist. In conclusion POT is unipolar when the proximal omentum remains fixed and the other tongues are free. SOT is bipolar due to a fixation of the omental tongue both proximally to the colon and distally, subsequently to adhesions for pathological conditions.

## Case Report

S. C., a retired man, aged 83, was admitted to our Hospital because of an intra-abdominal pain started a few days before in the absence of fever, vomiting or nausea. The patient felt a dull pain in right side of the abdomen for one day, he did not sleep during the subsequent night, then he was visited at home by his Practitioner who treated the patient pharmacologically. In the same day, when the abdominal pain became steady and dull, the patient was brought to First Aid Service of our University General Hospital. The Patient was affected by glaucoma, hypertension had a pace maker and had received right saphenectomy and right eye cataract interventions ten years before. At physical examination the abdomen appeared bloated, tenderly, with slow peristalsis, last evacuation the day before. There was moderate rigidity of the upper right side of the abdomen, with tenderness in the right and in the lower quadrants. At the admittance laboratory findings showed white blood cells count 7,640/mmc with 81.6% polymorphonuclear cells, increasing at the next days evaluations (91.6% polymorphonuclear cells), alfa-1 seroproteins 10.8 g/dl and glycaemia 173 mg/dl. As this disease may mimic other surgical emergencies, extensive imaging studies were performed.

**Figure 1:**
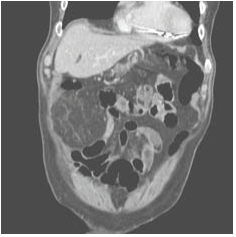
**Computerized tomography (CT) scan shows a characteristic fat pattern**. The vascular pedicle extends caudally and enters a large well-circumscribed heterogeneous fatty mass in the right lower quadrant and increased fat density.

**Figure 2:**
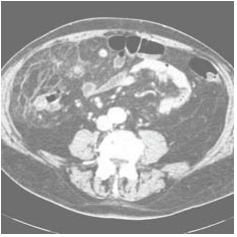
**Computerized tomography (CT) scan shows the fat pattern**. An omental vascular structure is seen at the center of concentrically layered streaks.

• Ultrasonography (US), which gave negative result,

• Computerized Tomography (CT) (Figure 1, 2) scan which showed an inhomogeneous, irregular edge profile mass of 38 × 30 × 25 cm of great omental appearance, localized at the right side, moreover, concentric distribution of fibrous and fatty folds converging radially toward the torsion with oedema of the fat tissue was evidenced.

**Figure 3:**
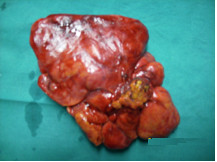
**Example of POT**. A normal appearing omentum was above the torsion point. You can see the vascular hanged and the torsion point with the distal thickened and congested omentum.

The operation was performed on the third day after admittance, in improved metabolic conditions. After a healthy appendix was found, we did inspect the cecum for a perforated diverticulum, the terminal ileum was then examined for Meckel diverticulum and regional enteritis, the pelvic organs were inspected and palpated, gallbladder and duodenum were observed, mesentery was evaluated for mesenteric lymphadenitis, no peritoneal pedicle torsion was found inside the umbilicus. Then we did observe the "omental ball" of 18 × 13 × 6 cm in size, which was suspended by a pedicle twisted on its axis four times (Figure 3) and was easily resected. Our patient had an uneventful recovery and was discharged two days after. **Histology Findings**: omental pedicle of 18 × 13 × 6 cm in size, with lobed and cyanotic look. Evidence of hemorrhagic infarction and focal fat necrosis areas at the section. **Microscopic**: Omental tissue characterized by acute extensive hemorrhagic infarction and fat necrosis, with polymorph nuclear cells infiltration of vein vessels and focal necrosis areas.

## Discussion

POT is a rare pathological condition which presents with generic symptoms, and may mimic a variety of acute abdominal conditions such as cholecystitis, acute diverticulitis, appendicitis [6] and Meckel diverticulum [7]. The pathogenesis of POT with infarction has not been established, however, some anatomical malformations and anomalies are recognized as predisposing factors to OT: presence in the great omentum of tongue-like projections and bifid and accessory omentum, anomalous vascular blood supply, other vascular anomalies that modify the weight of the omentum, vascular kinking, irregular omental pad, mostly in obese patients [8,9]. SOT is more common than POT and is associated with pre-existing abdominal pathologies, including cysts, tumours, foci of intra abdominal inflammations [10] and surgical wounds or scarring and hernial sacs [11]. Most cases of SOT occur in patients with inguinal hernia as reported by Morris et al. [2]. Mentioned in literature as precipitant factors are trauma of the abdominal wall, coughing, effect of lifting, bicycle racing, hard labour, ingestion of heavy meals, hyperperistalsys, violent purgation or the taxis of an hernia, causes of passive displacement of the omentum [12]. The OT determines the omentum twists around a pivotal point, usually in a clockwise direction. Engorgement of the tortuous veins that are more easily compressed may compromise venous return, and the distal omentum becomes congested and oedematous. Recovery may follow or the process may go on [2]. Resultant hemorrhagic extravasations create a characteristic serosanguineous fluid inside the great omentum and in the peritoneal cavity. As the torsion progresses, arterial occlusion leads to acute hemorrhagic infarction and eventual necrosis of the omentum occur [6]. Furthermore omental infarction can occur without torsion due to hypercoagulable state or vascular abnormalities predisposing to thrombosis [7]. Vasculitis or congestion of mesenteric veins may be caused by right sided heart failure [13,14]. The differential diagnosis between POT and SOT is difficult and has seldom been made during the operation. Helpful is US or CT scan. Usually US findings are evaluated as normal [7]. Some times US may show a complex mass or a mixture of solid material and hypoechoic zones. US is a diagnostic procedure useful to exclude other acute abdominal conditions. CT scan is an effective procedure in diagnosis of acute abdominal torsion [15-17]. Preoperative US or CT scan are mandatory and the preoperative diagnosis can be accurately accomplished by these procedures. With increased use of US and CT scan, preoperative diagnosis of POT may increased in frequency [18] and in selected cases can avoid surgery and lead to conservative treatment [19-21]. In practice, US and CT scan are often avoided only for economical reasons. CT scan of our patient showed an inhomogeneous irregular edge profile mass of 38×30×25 cm of omental appearance localized at the right side. Concentric distribution of fibrous and fatty folds converging radially toward the torsion with oedema of the fat tissue, of the mesentery and little fluid collection between the right muscle wall and the lower liver surface were shown. The same pattern of concentric linear streaks in the omental fat with high-attenuated vascular structure of omentum running perpendicular to the axial plane at the centre of a concentrically layered streaks was observed by Sakamoto et al. [22]. In their report, CT scan showed also a closed vascular pedicle. Balthazar et al. [15] showed effective also the MRI specially when OT is complicated by bleeding or development of an abscess [15]. Conversely, the radiography studies are ineffective in differential diagnosis between infarction of great omentum and infarction caused by torsion [9]. OT is usually diagnosed during explorative laparotomy that represents diagnostic and therapeutic procedure. Thus, laparoscopy is the first choice procedure for diagnosis and treatment of acute omental torsion [23]. This procedure permits definitive diagnosis, when US and imaging (CT and MRI) findings are unclear [24]. In all cases laparoscopy permits a correct diagnosis of omental infarction and surgical excision [25]. The minimally invasive access to the abdominal cavity without surgical incision evocates less pain than traditional procedure and permits a praecox discharge of the patient in the first postoperative day [26]. Furthermore, in cases of POT with extensive mass of omentum, the laparoscopic technique alone might require to long surgery time; in such cases the therapeutic management of choice is diagnostic laparoscopy proceeding to laparotomy [18], which can permit the omental excision with small abdominal incision.

## Conclusions

POT is a rare pathological condition with generic symptoms that may mimic many acute abdominal conditions. The pathogenesis of POT has not been established. Anatomical malformations and vascular anomalies are predisposing factors.

SOT is a more common condition than POT, due to pre-existing abdominal pathology: cysts, tumours, abdominal inflammatory foci, postsurgical wounds and hernial sacs.

The symptoms and the laboratory findings of POT are not specific and mimic other pathological abdominal conditions, for these reasons they make loose time to make diagnosis and provoke increasing degree and duration of OT.

The differential diagnosis between POT and SOT is difficult and has seldom been made during the surgical operation. Helpful are US and/or CT scan. MRI can be effective when OT is accompanied by infarction or abscess.

Explorative laparotomy represents a diagnostic and definitive therapeutic procedure.

Nowadays laparoscopy is the first choice procedure for diagnosis and treatment of acute abdominal torsion.

In cases of POT with extensive mass of omentum, diagnostic laparoscopy followed by laparotomy could permit the omental excision with small abdominal incison.

## Competing interests

The authors declare that they have no competing interests.

All authors read and approved the final manuscript.

## Authors' contributions

JA drafted the manuscript and participated in the management of patient care. CC carried out a revision of the literature about the topic. OM participated in the management of patient care. MC contributed to write down the manuscript and participated in the management of patient care. NP reviewed the manuscript. DT reviewed the manuscript, carried out the surgery and participated in its design and coordination. All authors read and approved the final draft.

## Consent

The patient knew about this case report and he signed a consent statement. A copy of the written consent was in the patient medical record.
